# Highly Pathogenic Avian Influenza A(H5N1) Virus Clade 2.3.4.4b Infections in Seals, Russia, 2023

**DOI:** 10.3201/eid3010.231728

**Published:** 2024-10

**Authors:** Ivan Sobolev, Alexander Alekseev, Kirill Sharshov, Maria Chistyaeva, Alexander Ivanov, Olga Kurskaya, Olesia Ohlopkova, Alexey Moshkin, Anastasiya Derko, Arina Loginova, Mariya Solomatina, Alimurad Gadzhiev, Yuhai Bi, Alexander Shestopalov

**Affiliations:** Federal Research Center of Fundamental and Translational Medicine, Novosibirsk, Russia (I. Sobolev, A. Alekseev, K. Sharshov, M. Chistyaeva, O. Kurskaya, O. Ohlopkova, A. Moshkin, A. Derko, A. Loginova, M. Solomatina, A. Shestopalov);; Green Sakhalin Nature and Environment Protection Fund, Kholmsk, Russia (A. Ivanov);; Dagestan State University, Makhachkala, Russia (A. Gadzhiev);; Chinese Academy of Sciences, Beijing, China (Y. Bi)

**Keywords:** influenza, influenza A, HPAI virus, seals, outbreak, H5N1, clade 2.3.4.4.b, mass death, Sea of Okhotsk, Tyuleniy Island, Russia, China, respiratory infections, zoonoses, viruses

## Abstract

Highly pathogenic avian influenza A(H5N1) virus was detected in dead seals on Tyuleniy Island in eastern Russia, in the Sea of Okhotsk. Viruses isolated from dead northern fur seals belong to clade 2.3.4.4b and are closely related to viruses detected predominantly in the Russian Far East and Japan in 2022–2023.

In July 2023, the deaths of northern fur seals (*Callorhinus ursinus*) and Steller sea lions (*Eumetopias jubatus*) were noted in the Far East region of the Russian Federation on Tyuleniy Island (Figure 1). The island is situated in the southwestern part of the Sea of Okhotsk, the northern part of the Pacific Ocean, close to Sakhalin Island. Tyuleniy Island has an area of 0.054 km^2^ and is devoid of water, woody vegetation, terrestrial predators, and permanent human settlements. Its unique environment enables marine mammals to form extensive rookeries and seabirds to establish nesting colonies (*1*), reaching extremely high densities of animals of different ages (Appendix 1 Figures 1–3). The population size of the northern fur seal on Tyuleniy Island in 2022 was ≈55,221 (*2*).

**Figure 1:**
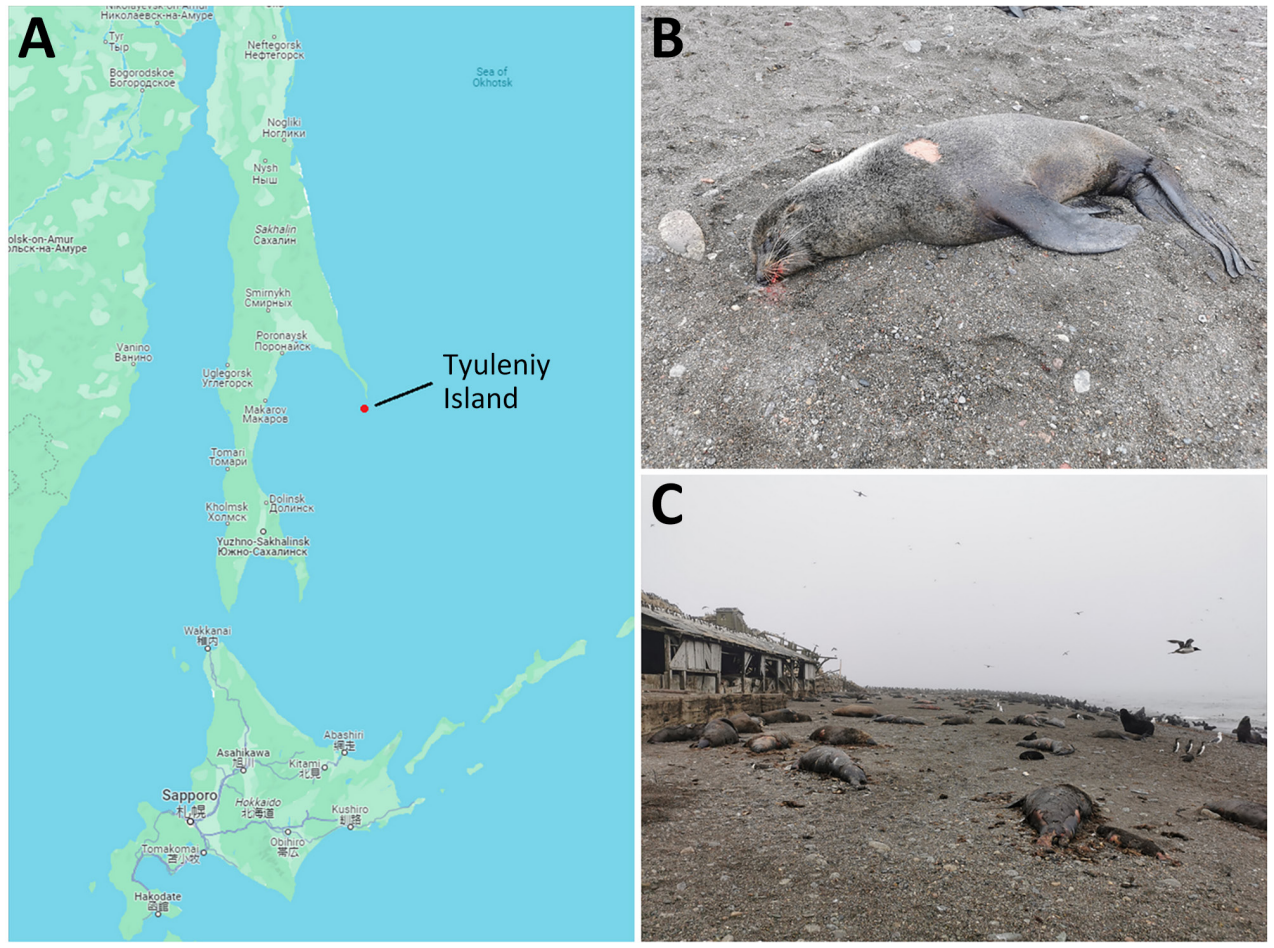
Investigation of seal deaths caused by highly pathogenic avian influenza A(H5N1) virus clade 2.3.4.4b on Tyuleniy Island in eastern Russia. A) Location of Tyuleniy Island in the Sea of Okhotsk. B, C) Deceased seals infected with the virus.

## The Study

We detected the first seal death on July 15, 2023, and a mass death of seals during July 15–August 15, 2023; a total of 3,500 northern fur seals and 1 Steller sea lion died. Many adult animals died in the surf or water; thus, it is likely that the actual number of animal deaths exceeds the number we counted. We found dead pups (1–5 weeks old) on August 4; pup deaths became widespread. In observing diseased animals, we identified 2 stages of disease progression from the onset of symptoms. In stage 1, lasting 6–8 hours, animals experienced fever, lethargy, confusion, and disorientation, and in stage 2, lasting 2–4 hours, they experienced convulsions and death.

We took samples from the lungs, small intestine, and liver of 2 deceased northern fur seals. We detected influenza A virus (IAV) of the H5 subtype in the lungs and small intestine of 1 animal and in the lungs and liver of the other animal by real-time PCR. We isolated IAV from the PCR-positive organs in embryonated chicken eggs. We sequenced whole genomes of 3 viruses isolated from the small intestine and lungs of the first animal and from the lungs of the second animal (Table) using Illumina MiSeq (https://www.illumina.com). We identified all isolates as highly pathogenic avian influenza (HPAI) viruses on the basis of the amino acid sequence of the hemagglutinin (HA) polybasic proteolytic cleavage site (PLREKRRKR/G) and intravenous pathogenicity index values of 2.90 in chickens. We determined the subtype of the HPAI virus neuraminidase (NA) through NA sequence analysis as N1.

**Table:** Highly pathogenic avian influenza viruses isolates from 2 northern fur seals on Tyuleniy Island, Russia, 2023*

Isolate name	Host	Source	GISAID ID
A/Northern_fur_seal/Russia_Tyuleniy/74/2023	Seal 1	Small intestine	EPI_ISL_18554237
A/Northern_fur_seal/Russia_Tyuleniy/74-2/2023	Seal 1	Lung	EPI_ISL_19080209
A/Northern_fur_seal/Russia_Tyuleniy/75/2023	Seal 2	Lung	EPI_ISL_19080210

*Samples were collected on August 10, 20203. GISAID, https://www.gisaid.org; ID, identification.

**Figure 2:**
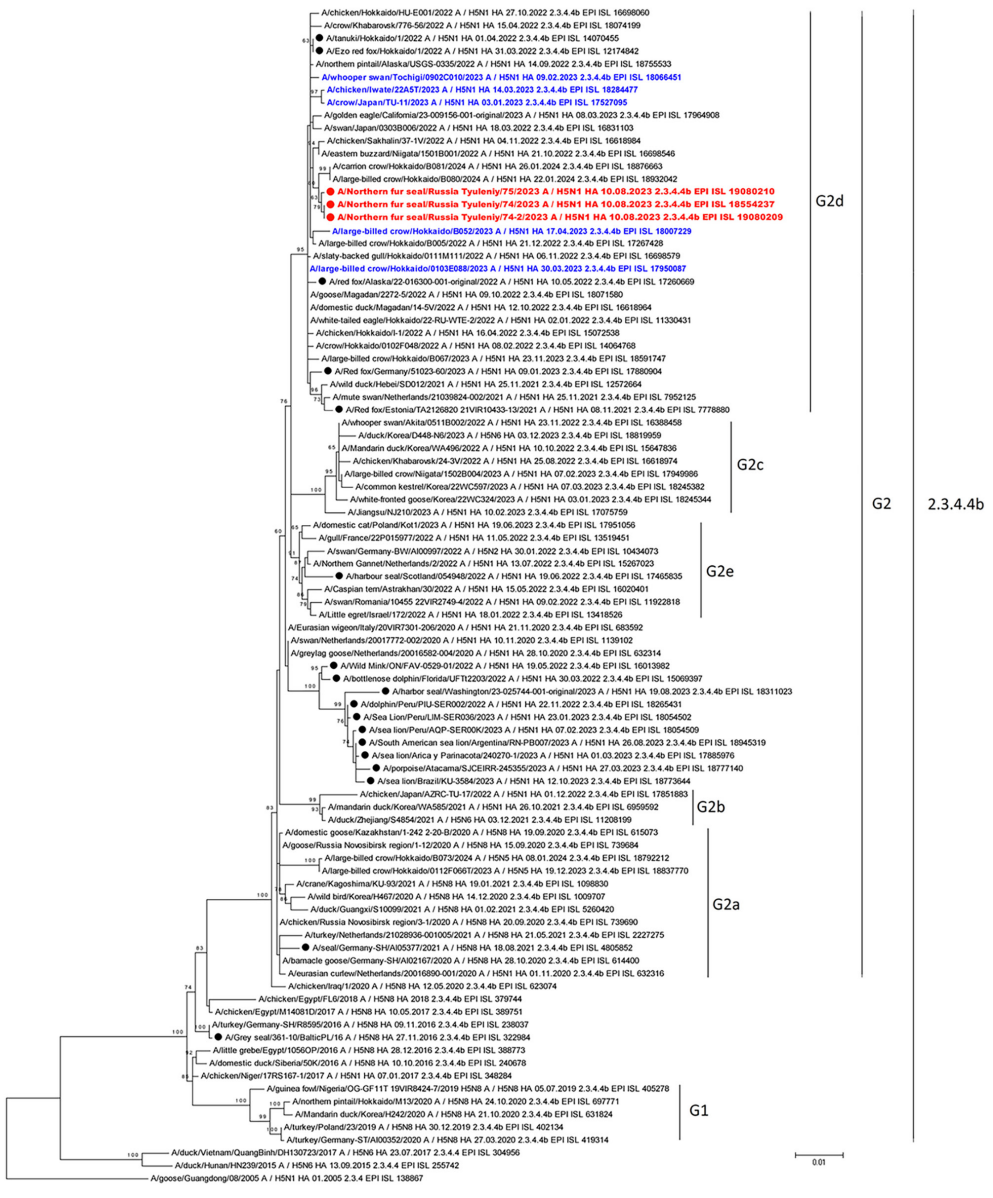
Maximum-likelihood phylogenetic tree of the hemagglutinin segment of HPAI A(H5N1) from northern fur seals on Tyuleniy Island in eastern Russia (red) and in other marine mammals, by clade group. Black dots indicate HA of viruses isolated from other mammals, and blue text indicates HPAI viruses isolated in Japan during January‒April 2023. GISAID accession numbers are shown for reference sequences (https://www.gisaid.org). Note that dates are shown in DD.MM.YYYY format. Scale bar indicates number of substitutions per site. HA, hemagglutinin; HPAI, highly pathogenic avian influenza.

Phylogenetic analysis of the HA segment revealed that the strains isolated from northern fur seals on Tyuleniy Island belonged to HPAI H5N1 virus clade 2.3.4.4.b of the A/goose/Guangdong/1/96-like (Gs/GD) lineage (Figure 2). We found the HA segments of the viruses isolated from northern fur seals on Tyuleniy Island belonged to G2 group of clade 2.3.4.4b (Figure 2). Clade 2.3.4.4.b was divided into groups G1 and G2. Subsequently, several subgroups were identified in group G2: G2a–G2e (*3*,*4*). The G2 group comprises viruses detected in Egypt during 2017‒2019, in Iraq in May 2020, in Russia, Kazakhstan, and Europe in July–November 2020 (*5**–**8*) and subsequently spread throughout Eurasia (*3*,*4*).

Whole-genome phylogenetic analysis (Figure 2; Appendix 1 Figures 4‒10) revealed that viruses from northern fur seals are closely related to HPAI viruses circulating predominantly in Japan (in January–March 2022, October‒December 2022, and January‒April 2023) and to viruses detected in the Far East of Russia in April and October‒November 2022. HPAI viruses circulating in Japan (September 2022‒April 2023 and November 2023) and in the Far East of Russia, Khabarovsk and Magadan, in April 2022 and in October 2022, belong to 3 genetic subgroups (G2b, G2c, and G2d) of clade 2.3.4.4b (*9*). Viruses from Tyuleniy Island clustered with G2d subgroup viruses described earlier by Hew et al. (*9*). Phylogenetic and BLAST analysis (https://blast.ncbi.nlm.nih.gov) of HA sequences of H5Nx influenza viruses detected in mammals indicated that the viruses from seals from Tyuleniy Island are related to other mammal viruses isolated from raccoon dogs and Ezo red foxes in Japan in 2022, as well as from red foxes in Estonia in 2021 and Alaska in 2022. Previously published literature and information from GISAID EpiFlu (https://www.gisaid.org) showed multiple instances of HPAI H5Nx clade 2.3.4.4b virus infection in mammals, including marine mammals (*10*–*14*).

During 2022–2023, the HPAI H5 virus spread extensively throughout Japan, affecting a wide range of hosts, including not only wild waterfowl but also poultry and birds of prey. The virus was also detected in the Russian Far East, particularly in Magadan and Sakhalin. Data obtained from satellite tracking (*15*) showed that birds wintering in Japan mainly migrate in the spring to Kamchatka, often via Sakhalin, and then further to Chukotka. Some birds remain on Sakhalin, and a small number migrate to the Kuril Islands and Kolyma (and further to Chukotka).

The coexistence of birds and marine mammals on Tyuleniy Island creates favorable conditions for the spread of viruses, not only among birds but also for their transmission to seals during the breeding season (April‒August). On the basis of phylogenetic analysis, the timing of outbreak, and data on bird migration in the Pacific region, we suggest the virus that caused the deaths of marine mammals on Tyuleniy Island near Sakhalin in 2023 likely entered their populations as a result of spring bird migration from Japan. Viruses isolated from 2 northern fur seals on Tyuleniy Island are closely related but not completely identical. They differed by several amino acid substitutions, including the E627K mutation in PB2 of strains A/Northern fur seal/Russia_Tyuleniy74/2023 and A/Northern fur seal/Russia_Tyuleniy74-2/2023 isolated from the small intestine and lungs of 1 animal. That mutation is known to be associated with a shift in virus host specificity from birds to mammals and has been repeatedly detected, including in viruses isolated from marine mammals (*13*,*14*).

## Conclusions

The results of our study, combined with information from databases and data published in the literature, show that HPAI viruses have been repeatedly detected in mammals, including marine mammals, for several years. GISAID contains the nucleotide sequences of 84 strains of HPAI virus isolated from seals and sea lions. Most of the reported cases were identified during 2021‒2023 and were caused by HPAI H5Nx viruses of clade 2.3.4.4.b.

The widespread distribution of this relatively new variant of the influenza virus has resulted in cases of transmission from birds to mammals. It is likely that the high population density of birds and marine mammals on the small Tyuleniy Island, as well as their close proximity, contributed to the widespread infection of seals with HPAI virus. Transmission from birds probably occurred in 2 ways: through adult seals eating sick or dead birds, and through seal adults’ and pups’ contact with feces from infected birds. Unfortunately, the variability and transmission of the detected virus cannot be traced without a long-term study of the virus in parallel in the Tyuleniy Island bird population and in the seal population, starting from the beginning of spring bird migration. Even so, the number of seal deaths, mass mortality of pups, phylogenetic differences with viruses circulating in October‒December 2022 and January‒April 2023, and the detection of the E627K substitution indicate the possibility of adaptation and transmission from seal to seal.

Appendix 1 Additional information about highly pathogenic avian influenza A(H5N1) in marine mammals in eastern Russia, 2023.

Appendix 2 Data obtained from GISAID for study of highly pathogenic avian influenza A(H5N1) in marine mammals in eastern Russia, 2023.
