# Supplementary material for: Highly Pathogenic Avian Influenza A(H5N1) Virus Clade 2.3.4.4b Infections in Seals, Russia, 2023
Source: Emerg Infect Dis. 2024 Oct;30(10):2160–4. doi: 10.3201/eid3010.231728 (PMC11431915; doi:10.3201/eid3010.231728)
Supplement: Appendix 1 — Additional information about highly pathogenic avian influenza A(H5N1) in marine mammals in eastern Russia, 2023. [file 23-1728-Techapp-s1.pdf]

Article DOI: http://doi.org/10.3201/eid3010.231728

EID cannot ensure accessibility for supplementary materials supplied by authors. Readers who have difficulty accessing supplementary content should contact the authors for assistance.

Highly Pathogenic Avian Influenza A(H5N1) Virus Clade 2.3.4.4b Infections in Seals, Russia, 2023

Appendix 1

Materials and Methods

Location, Ecology and Sample Collection

Tyuleniy Island is home to the hugest bird colony in the Far East and one of the hugest in the world. 143 bird species were found on the island (*1*), the Common Murre predominates, with over 145,000 individuals in 2019. Also, the photo (Figure 1; Appendix Figures 1–3) shows that birds not only fly over the rookery of seals but also walk among them.

There is a continuous natural death of birds on the island. Natural bird mortality mainly occurs in the central, inaccessible for seals part of the island. However, according to the description of Tyuleniy Island (*2*), there are abandoned structures (partially visible in Figure 1) and debris occupying a large part of the island. This often results in the death of birds and marine mammals as they climb into the structures and cannot get out, injuring themselves on the debris. In addition, sick and, consequently, sedentary birds may be found among seals both on land and in the water. Upon visual inspection of the coastal area of the island, dead birds were observed, some of which were found among seals.

One sexually mature female of northern fur seal gives birth to one pup. The breeding season of the northern fur seal begins at the end of April and continues until the start of August. At the end of April, the first adult male fur seals begin to arrive at the rookery on Tyuleniy Island. According to long-term observations, the first female seals arrive at the rookery in early June, and the last female seals come to the rookery in late July. Female fur seals come to the rookery pregnant and give birth a few hours or days later.

At the beginning of the outbreak, adult males aged 5–10 years were deceased, followed by the death of pups (both males and females). The pups were 1 to 5 weeks old. Female mortality was rarely recorded at rookeries. However, a large number of dead seals were found in the water, but their sex and age were not assessed. We assume that these were predominantly females, as unlike pups and males, they regularly leave the rookery to feed.

During the period of mass mortality of seals, a significant number of dead birds were observed in the coastal area where seals rookeries are located. The northern fur seal is an unspecialized predator, with a diet consisting of 50 species of fish from 7 families and 10 species of squid. On rare occasions, it may eat birds and crustaceans. Thus, birds are not a standard part of seals' diet, but they may be eaten if they are catchable or if the bird is dead. Additionally, the high number of birds on the island naturally leads to a high amount of their feces, contact with which may also result in infection.

The number of deceased animals was visually assessed during the island rounds by ecologists and volunteers. There are no rookeries of Steller's sea lions on Tyuleniy Island. It is not a breeding site of this animals, and there is no counting of these animals on the island. Visually, seven dead adult Steller's sea lions and three pups were found during the island walkover. No biologic material was collected from Steller sea lions. Thus, we found only 10 dead Steller's sea lions, which is a negligible number compared to all deceased mammals. No samples were obtained from them.

Samples were collected on August 10, 2023, from two young male northern fur seals (5 years old) at a rookery in the coastal zone of Tyuleniy Island. One animal was already found dead, but without any signs of decomposition. The second animal was observed during death, and sectional material was collected from it immediately after the animal was died.

During northern fur seals sample collection, the sectional material was frozen in liquid nitrogen. After delivery to the biosafety level 3 laboratory, samples were homogenized, and RNA isolation was performed using the RIBO-prep Total RNA/DNA isolation reagent kit (AmpliSens, Russia).

Virus Detection

In all of these samples, AIV type A and HA of H5 subtype were detected using multiplex real-time polymerase chain reaction (AmpliSens Influenza virus A H5N1-FRT PCR kit, AmpliSens, Russia). All viruses analyzed were isolated from 10-day-old chicken embryonating eggs using chicken embryo inoculation. All viruses caused the death of chicken embryos within 2 days. Isolates were demonstrated to be H5 positive using Real-Time qPCR.

Genome Sequencing and Phylogenetic Analysis

Complete genome NGS sequencing was performed using the Illumina MiSeq platform and associated reagent kits, also from Illumina, according to the manufacturer's methodology. RNA was extracted using the QIAamp Viral RNA Mini Kit (Qiagen, Germany). Whole-genome amplification was performed using the modified protocol presented by Bin Zhou (*3*). DNA libraries were prepared using a Nextera DNA Flex Library Prep kit (Illumina, San Diego, CA, USA). Sequencing of the DNA libraries was conducted with a reagent kit, version 3 (600-cycle), on a MiSeq genome sequencer (Illumina). The consensus sequences were generated using Bowtie software.

Nucleotide sequences were deposited in the GISAID database (EPI_ISL_18554237, EPI_ISL_19080209, and EPI_ISL_19080210 (Table). The multiple alignment was performed using MUSCLE, and its editing, including translation of the nucleotide sequences to amino acid sequences, was performed using the BioEdit and UGENE software. Initial Maximum-likelihood phylogenies for each of the gene segments were generated with RAxML (*4*) using the general time-reversible nucleotide substitution model. Final dendrograms were generated and visualized with MEGA5 (*5*). Bootstrap support values were generated using 1,000 rapid bootstrap replicates.

Intravenous Pathogenicity Index (IVPI)

All aspects of the present study were approved by the Committee on Biomedical Ethics at the Federal Research Center of Fundamental and Translational Medicine (Novosibirsk, Russia). Animal experiments were conducted in biosafety level 3 facilities. For the determination of IVPI of 9 viruses, 0.1 ml of 1:10 dilution of infectious allantoic fluids were inoculated intravenously into ten 6-week-old specific pathogen free chickens. The IVPI was calculated according to the OIE standard protocol (available at: http://www.oie.int/international-standard-setting/terrestrial-

code/). Isolates with an IVPI >1.2 were determined to be HPAIV. The challenge study and all experiments with live viruses were conducted in a biosafety level 3 facility.

Limitations.

The main limitations of our study are related to the available primary material. In our work, we are limited to the field stage, which serves as the foundation of the entire study. Specifically, sectional samples were taken from only two northern fur seals. Lung, liver, and small intestine samples were taken, but not brain and trachea. There was no biologic material from Steller sea lions.

No biologic material has been collected from birds on Tyuleniy Island. Thus, it is difficult to draw any conclusions about the evolution of HPAI viruses in the ecosystem of Tyuleniy Island without extensive collection of samples from both marine mammals and birds, including feces.

References

1. Yusupova DA, Artemyeva SM, Kirillova AD, Nikishina AK, Vasukov ES, Red'kin YA, et al. Avifauna of Tuleny Island, Sea of Okhotsk [in Russian]. Ornitologia. 2021;45.

2. Shchelkanov MYu, Galkina IV, Ananiev VYu, Samarsky SS, Lienho VYu, Dedkov VG, et al. Ecological situation on the Tyuleniy Island in the Okhotsk Sea (2015): population interactions between pinnipeds, birds, ixodidae ticks and viruses [in Russian]. South of Russia: ecology, development. 2017;12(1):30–43. https://doi.org/10.18470/1992-1098-2017-1-30-43

3. Zhou B, Donnelly ME, Scholes DT, St George K, Hatta M, Kawaoka Y, et al. Single-reaction genomic amplification accelerates sequencing and vaccine production for classical and Swine origin human influenza a viruses. J Virol. 2009;83:10309–13. PubMed https://doi.org/10.1128/JVI.01109-09

4. Stamatakis A. RAxML version 8: a tool for phylogenetic analysis and post-analysis of large phylogenies. Bioinformatics. 2014;30:1312–3. PubMed https://doi.org/10.1093/bioinformatics/btu033

5. Tamura K, Peterson D, Peterson N, Stecher G, Nei M, Kumar S. MEGA5: molecular evolutionary genetics analysis using maximum likelihood, evolutionary distance, and maximum parsimony methods. Mol Biol Evol. 2011;28:2731–9. PubMed https://doi.org/10.1093/molbev/msr121

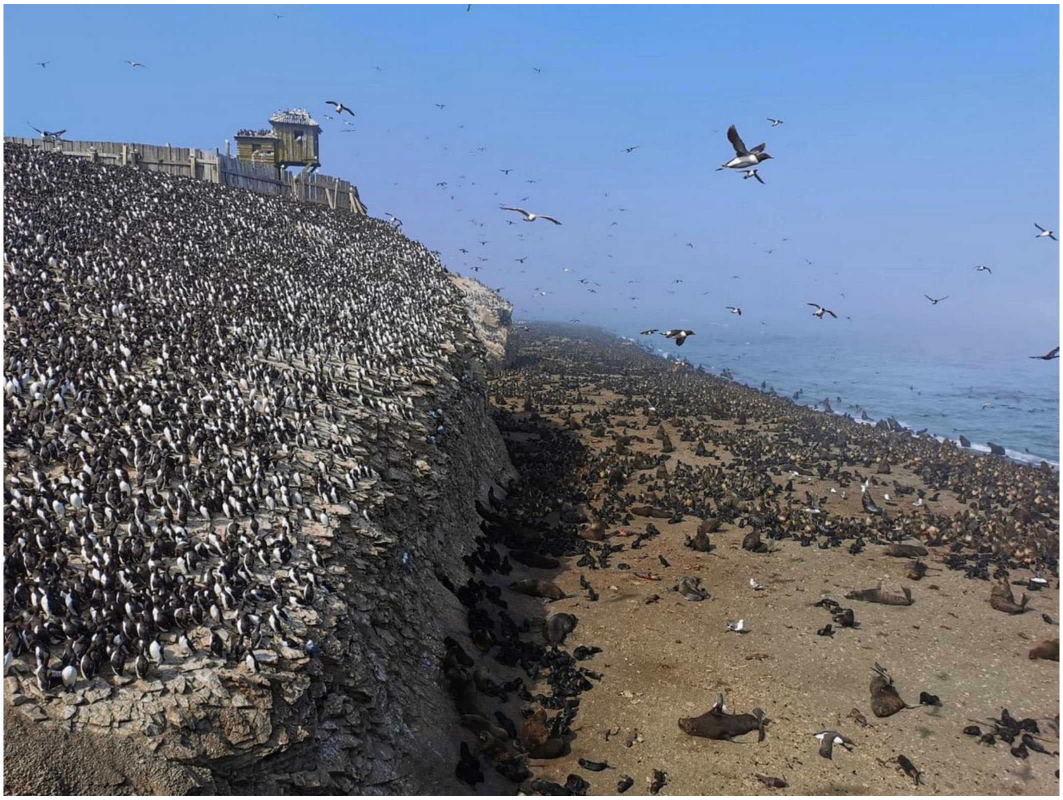

Appendix 1 Figure 1. Birds and seals of Tyuleniy Island.

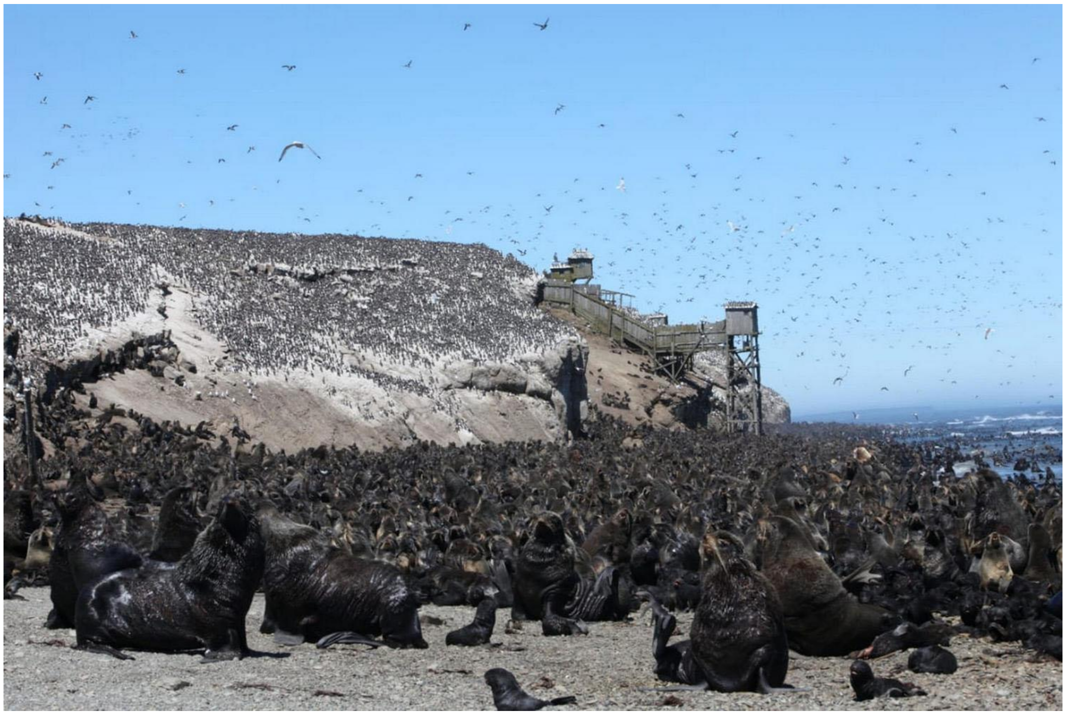

Appendix 1 Figure 2. Birds and seals of Tyuleniy Island.

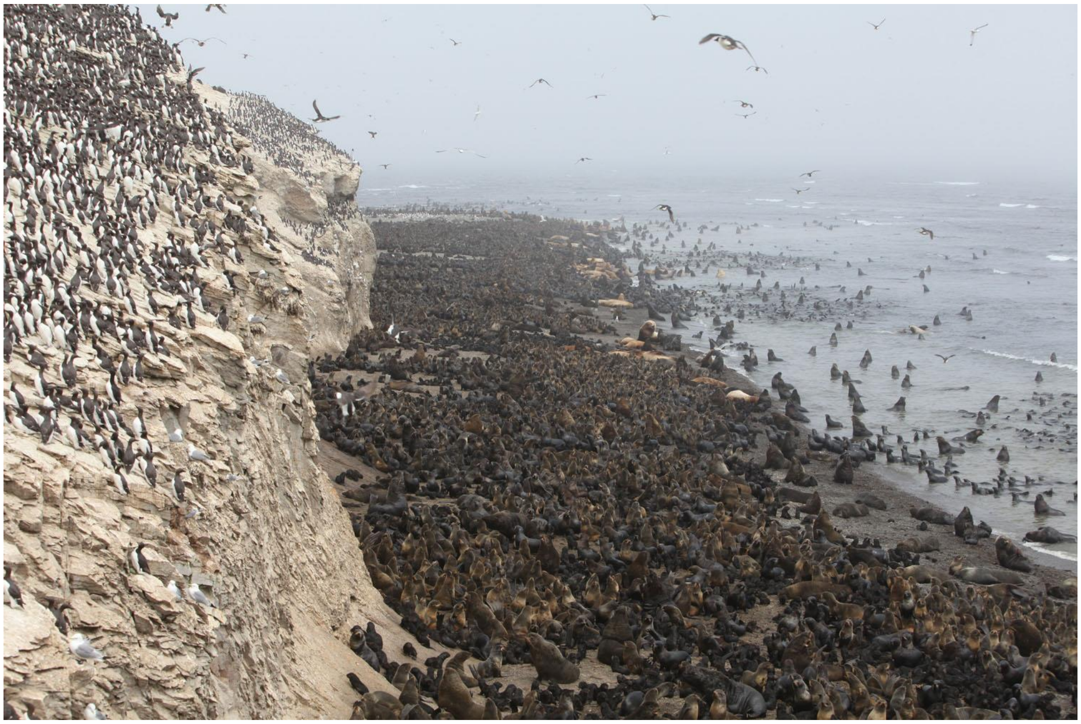

Appendix 1 Figure 3. Birds and seals of Tyuleniy Island.

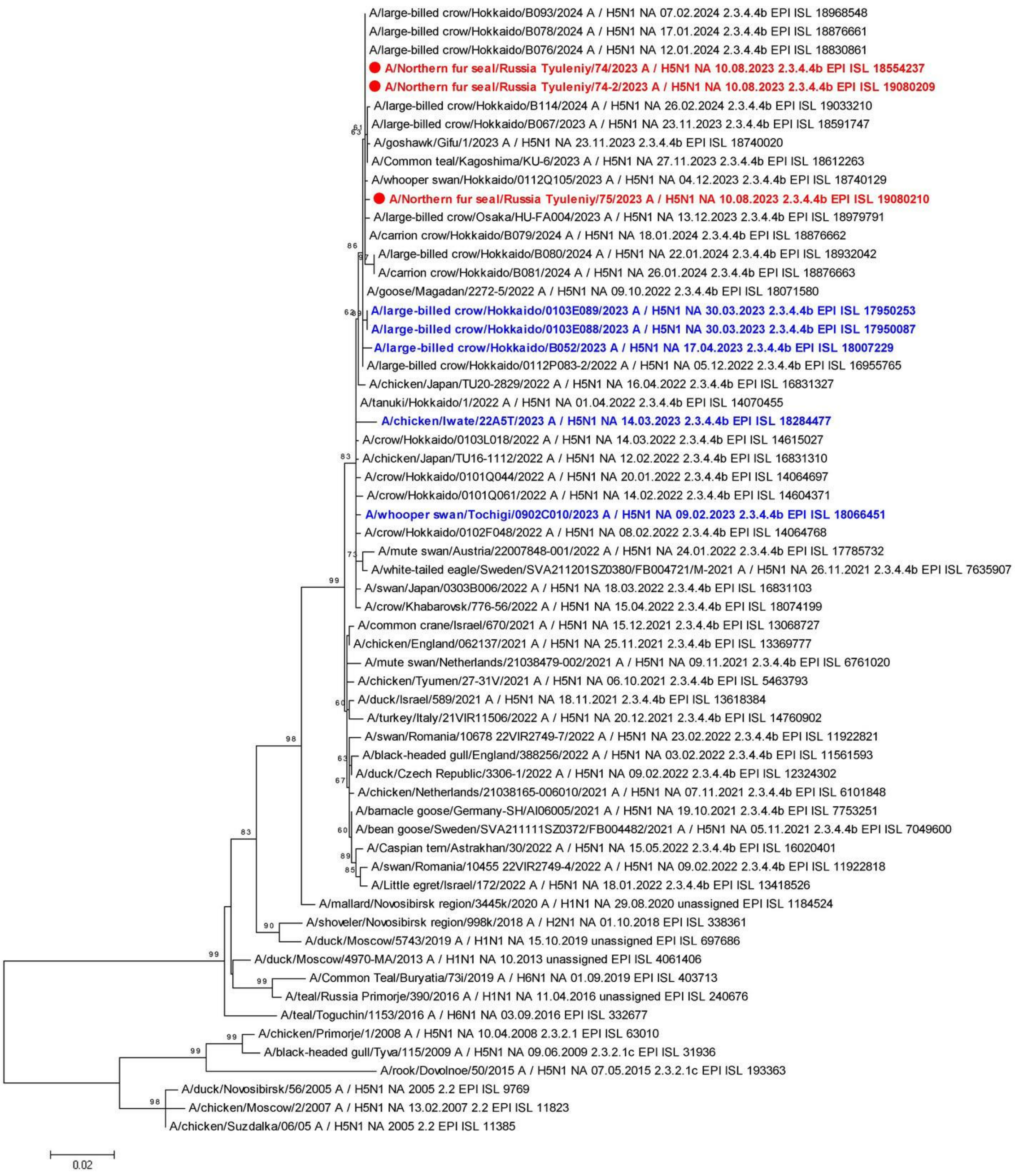

Appendix 1 Figure 4. Maximum-likelihood phylogenetic tree of the NA segment. Red markers and red font indicate NA of HPAI H5N virus strains from northern fur seals. Blue font indicates NA of HPAI viruses, isolated in Japan during winter/spring 2023.

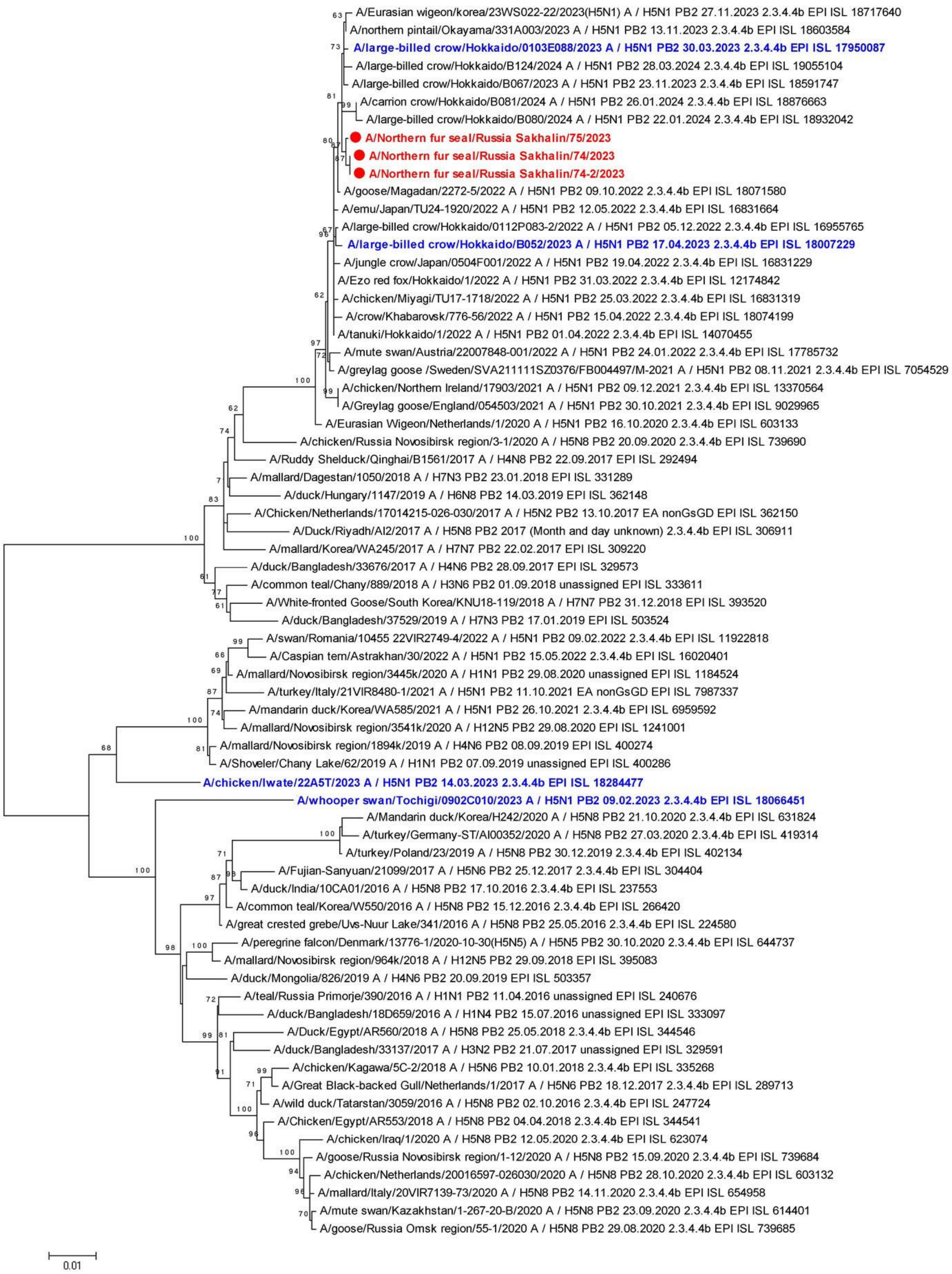

Appendix 1 Figure 5. Maximum-likelihood phylogenetic tree of the PB2 segment. Red markers and red font indicate PB2 of HPAI H5N virus strains from northern fur seals. Blue font indicates PB2 of HPAI viruses, isolated in Japan during winter/spring 2023.

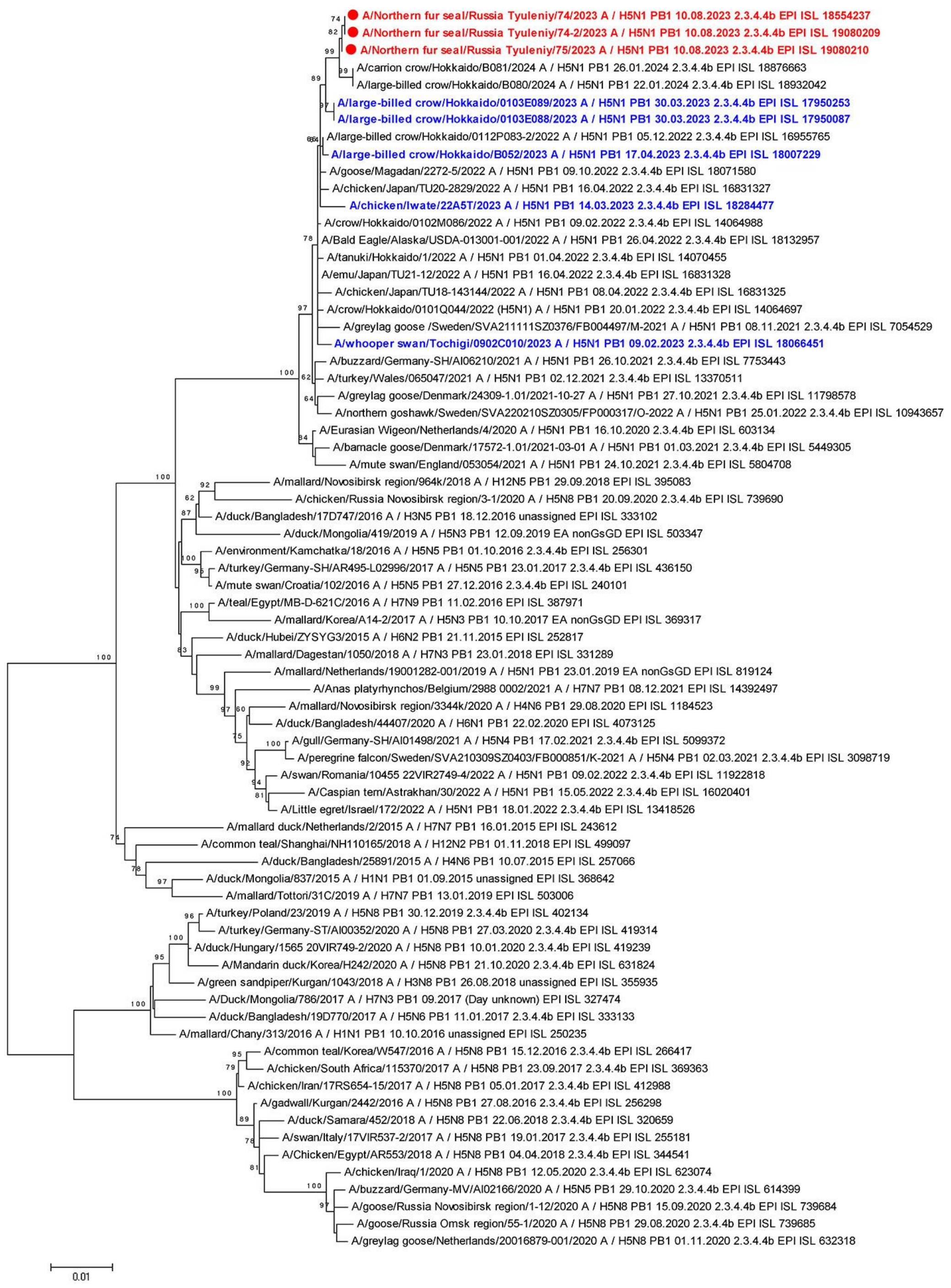

Appendix 1 Figure 6. Maximum-likelihood phylogenetic tree of the PB1 segment. Red markers and red font indicate PB1 of HPAI H5N virus strains from northern fur seals. Blue font indicates PB1 of HPAI viruses, isolated in Japan during winter/spring 2023.

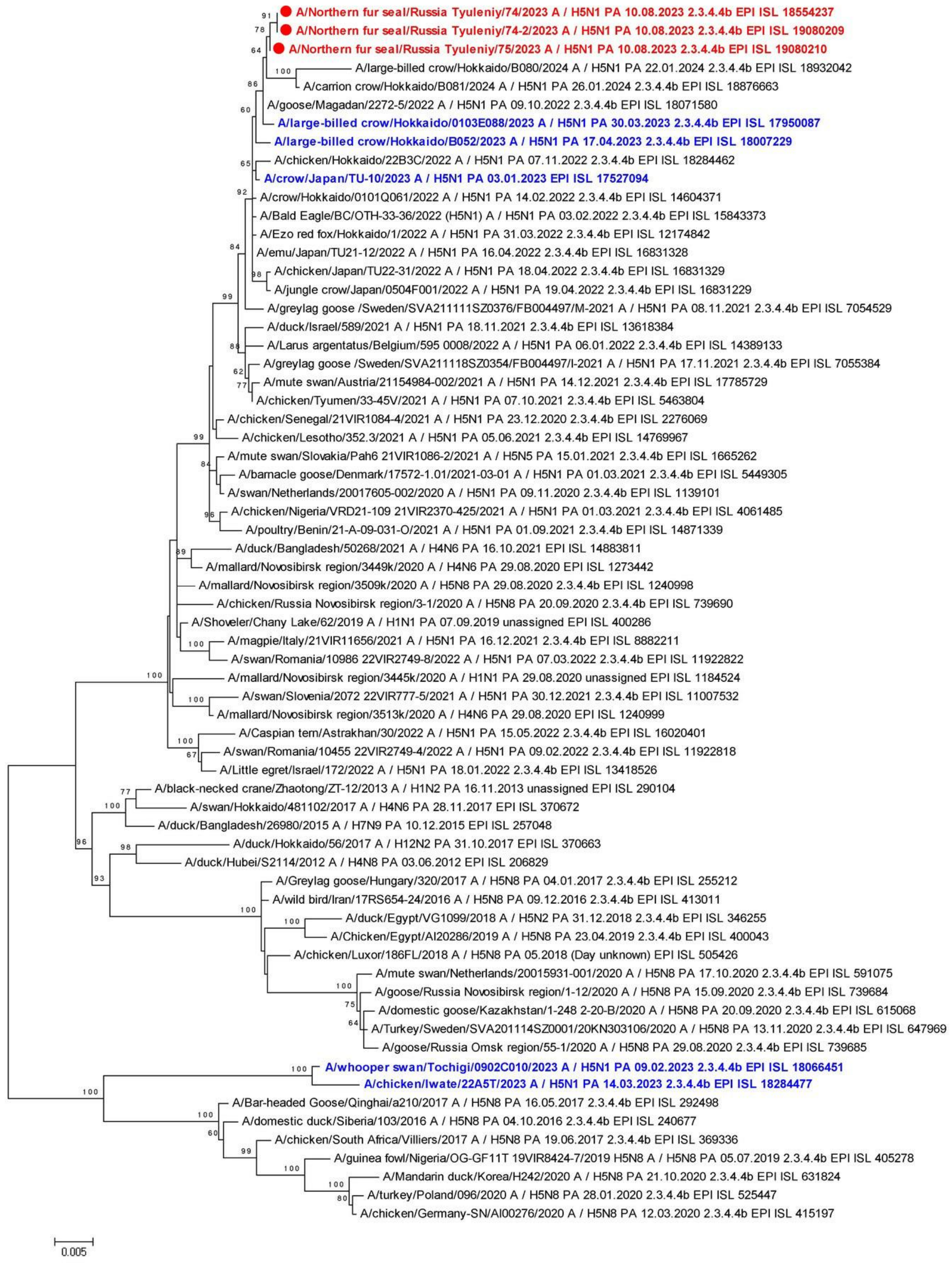

Appendix 1 Figure 7. Maximum-likelihood phylogenetic tree of the PA segment. Red markers and red font indicate PA of HPAI H5N virus strains from northern fur seals. Blue font indicates PA of HPAI viruses, isolated in Japan during winter/spring 2023.

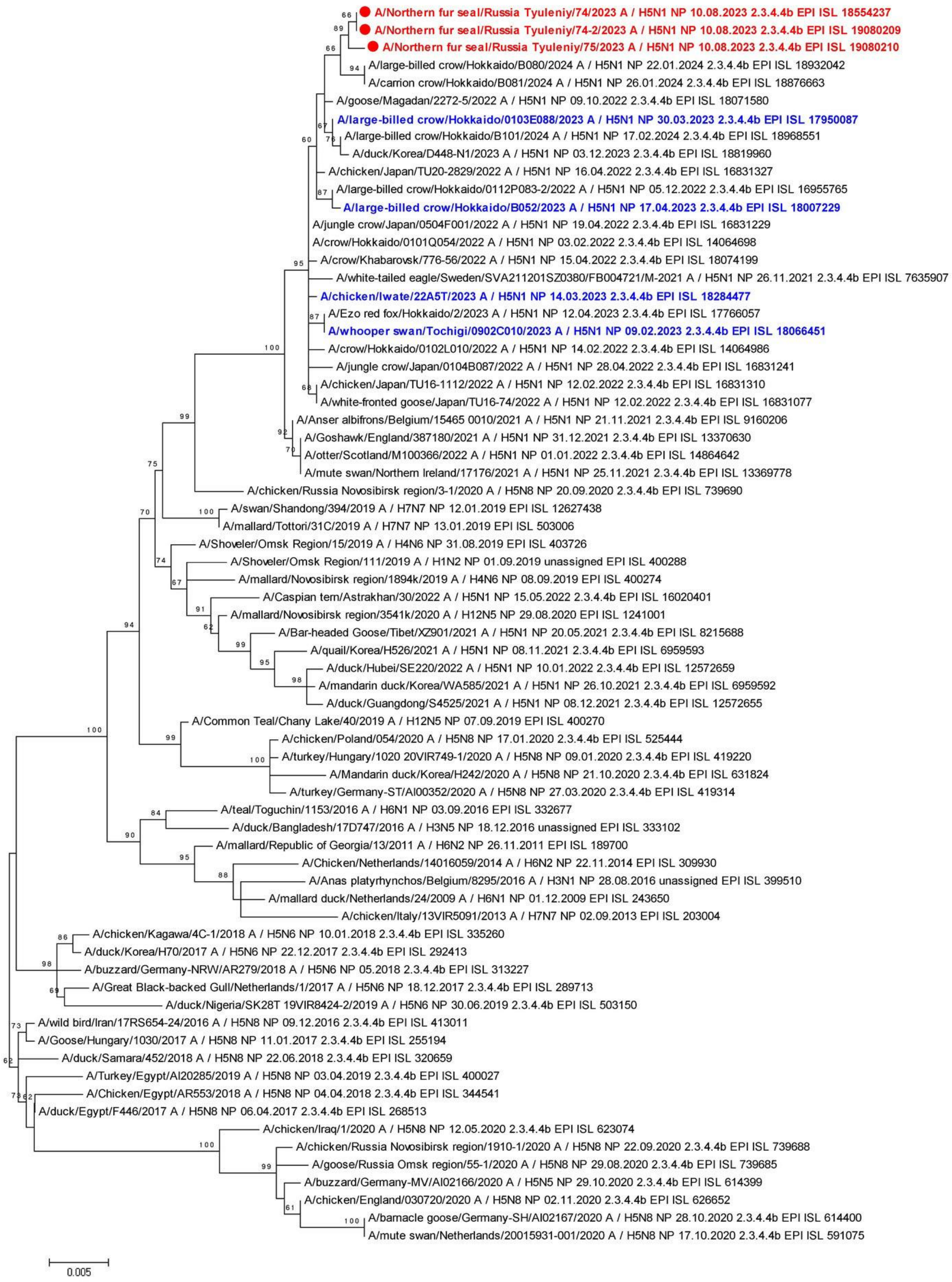

Appendix 1 Figure 8. Maximum-likelihood phylogenetic tree of the NP segment. Red markers and red font indicate NP of HPAI H5N virus strains from northern fur seals. Blue font indicates NP of HPAI viruses, isolated in Japan during winter/spring 2023.

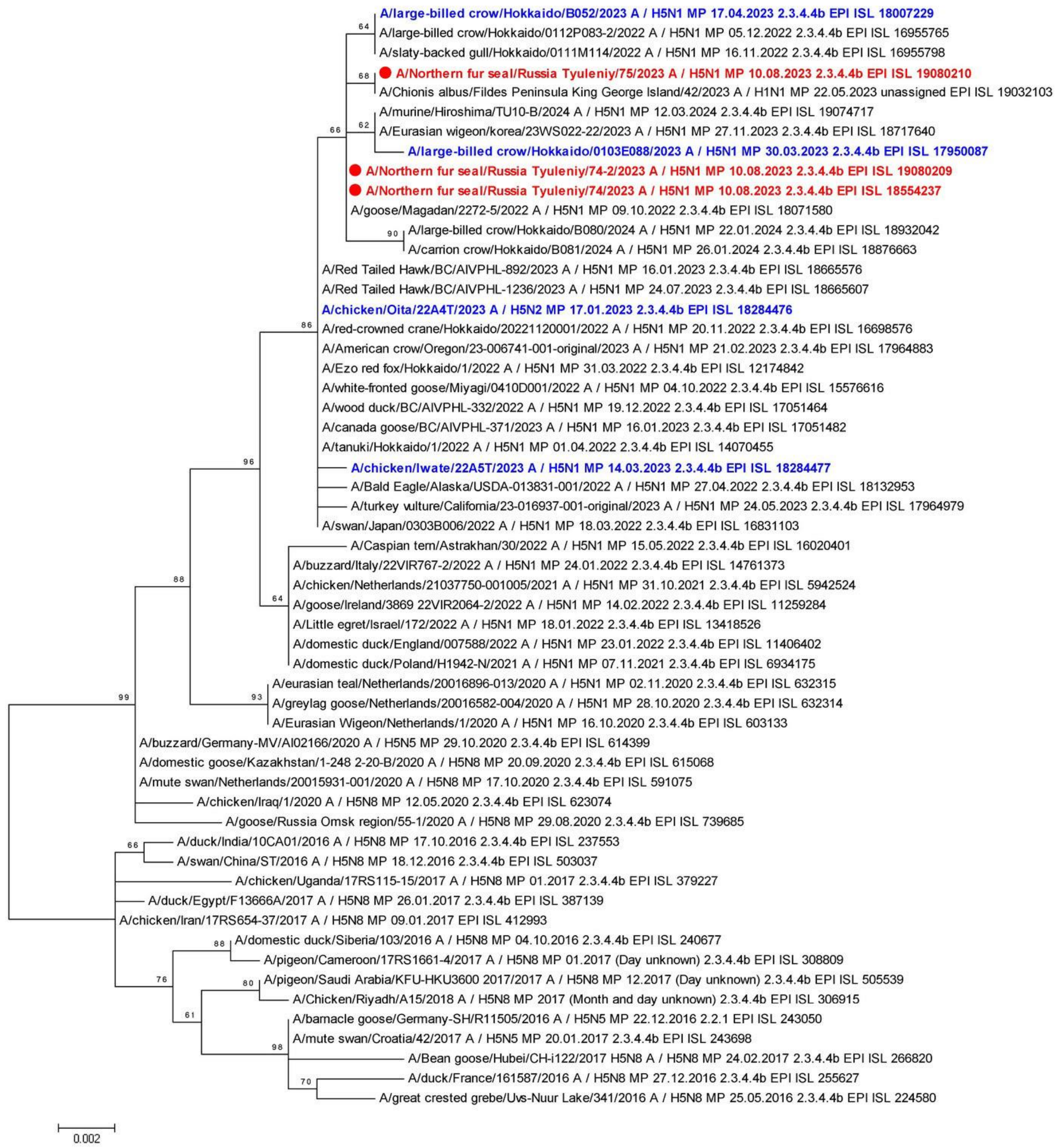

Appendix 1 Figure 9. Maximum-likelihood phylogenetic tree of the M segment. Red markers and red font indicate M of HPAI H5N virus strains from northern fur seals. Blue font indicates M of HPAI viruses, isolated in Japan during winter/spring 2023.

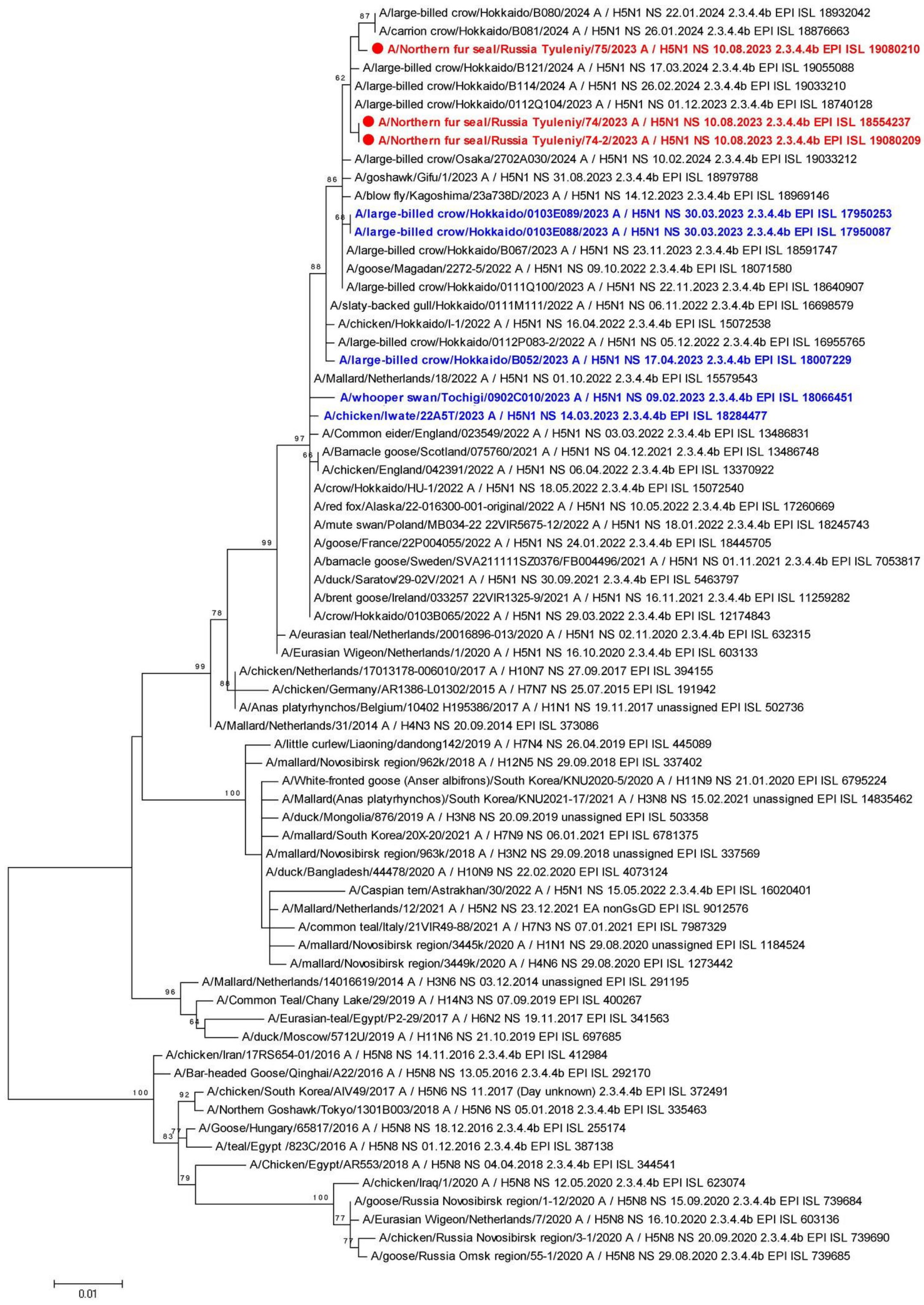

Appendix 1 Figure 10. Maximum-likelihood phylogenetic tree of the NS segment. Red markers and red font indicate NS of HPAI H5N virus strains from northern fur seals. Blue font indicates NS of HPAI viruses, isolated in Japan during winter/spring 2023.
